# Airway pathogens detected in stable and exacerbated COPD in patients in Asia-Pacific

**DOI:** 10.1183/23120541.00057-2022

**Published:** 2022-09-26

**Authors:** Laura Taddei, Lucio Malvisi, David S. Hui, Ludovic Malvaux, Ronnie Z. Samoro, Sang Haak Lee, Yiu Cheong Yeung, Yu-Chih Liu, Ashwani Kumar Arora

**Affiliations:** 1 GSK, Siena, Italy; 2 Dept of Medicine and Therapeutics, The Chinese University of Hong Kong, Shatin, Hong Kong SAR, China; 3 Stanley Ho Centre for Emerging Infectious Diseases, The Chinese University of Hong Kong, Shatin, Hong Kong SAR, China; 4 GSK, Wavre, Belgium; 5 Healthlink (Iloilo) Inc., Iloilo City, Philippines; 6 Division of Pulmonary, Critical Care and Sleep Medicine, Dept of Internal Medicine, Eunpyeong St Mary's Hospital, College of Medicine, The Catholic University of Korea, Seoul, Republic of Korea; 7 Dept of Medicine and Geriatrics, Princess Margaret Hospital, Kwai Chung, Hong Kong SAR, China; 8 Dept of Thoracic, Critical Care and Sleep Medicine, Chang Gung Memorial Hospital, Keelung, Chang Gung University, Taoyuan, Taiwan; 9 These authors contributed equally

## Abstract

**Background:**

The burden of chronic obstructive pulmonary disease (COPD) in the Asia-Pacific region is projected to increase. Data from other regions show bacterial and viral infections can trigger acute exacerbations of COPD (AECOPD).

**Methods:**

This 1-year prospective epidemiological study (ClinicalTrials.gov identifier: NCT03151395) of patients with moderate to very severe COPD in Hong Kong, the Philippines, South Korea and Taiwan assessed the prevalence in sputum samples (by culture and PCR) of bacterial and viral pathogens during stable COPD and AECOPD. The odds of experiencing an exacerbation was evaluated for pathogen presence, acquisition and apparition. Health-related quality of life (HRQOL) was assessed.

**Results:**

197 patients provided 983 sputum samples, with 226 provided during exacerbation episodes. The mean yearly AECOPD incidence rate was 1.27 per patient. The most prevalent bacteria by PCR at exacerbation were *Haemophilus influenzae* (Hi) and *Moraxella catarrhalis* (Mcat); Mcat prevalence was higher at exacerbation than at stable state. Virus prevalence was low, other than for human rhinovirus (HRV) (8.1%, stable state; 16.6%, exacerbation). The odds ratio (95% CI) for an exacerbation (*versus* stable state) was statistically significant for the presence, acquisition and apparition of Hi (2.20, 1.26–3.89; 2.43, 1.11–5.35; 2.32, 1.20–4.46, respectively), Mcat (2.24, 1.30–3.88; 5.47, 2.16–13.86; 3.45, 1.71–6.98, respectively) and HRV (2.12, 1.15–3.91; 2.22, 1.09–4.54; 2.09, 1.11–3.91, respectively). HRQOL deteriorated according to the number of exacerbations experienced.

**Conclusion:**

In patients with COPD in the Asia-Pacific region, the presence of Hi, Mcat or HRV in sputum samples significantly increased the odds of an exacerbation, providing further evidence of potential roles in triggering AECOPD.

## Abstract 2

**The presence of *Haemophilus influenzae, Moraxella catarrhalis* or human rhinovirus in sputum samples significantly increases the odds of an exacerbation, as opposed to being in stable state, in COPD** https://bit.ly/39vI3XU

## Introduction

Chronic obstructive pulmonary disease (COPD) is a common cause of morbidity and the third leading cause of mortality worldwide, accounting for 5.8% of all deaths [1, 2]. The burden of COPD is higher in the Asia-Pacific region than in industrialised Western countries [3]. Smoking and air pollution, either occupational or household from burning biomass, are among the main causes of COPD in the region [4–6].

Acute exacerbations of COPD (AECOPD) contribute to disease progression [1, 7, 8] and significantly impair patients’ quality of life [9]. Bacterial and viral infections are recognised as having an important association with AECOPD [7, 10, 11]. Various studies have shown a link between exacerbation and increased prevalence of airway bacteria, including *Haemophilus influenzae* (Hi) and *Moraxella catarrhalis* (Mcat) [12–18], and mixed bacterial and viral infections are frequent [11, 19–23]. In the observational Acute Exacerbation and Respiratory InfectionS in COPD (AERIS) study, bacterial and viral co-infection was more frequent during an exacerbation than at stable state [17]. Moreover, the odds of experiencing an exacerbation (*versus* stable state) were higher when both non-typeable Hi and human rhinovirus (HRV) were detected than when HRV was absent.

There are few data from the Asia-Pacific region on the prevalence of bacterial and viral infections during periods of stable and exacerbated COPD [24]. We conducted a study to estimate the prevalence of bacterial and viral pathogens, overall and by species, with an emphasis on Hi and Mcat, in patients with moderate to very severe COPD in selected Asia-Pacific populations. The incidence rate and duration of AECOPD was estimated, as well as the odds of experiencing an exacerbation in the presence of specific pathogens. The impact of AECOPD on health-related quality of life (HRQOL) was also calculated for the study population.

## Methods

### Study design, setting and objectives

This was a prospective epidemiological study (ClinicalTrials.gov identifier: NCT03151395) of patients with moderate to very severe COPD. All enrolled patients provided written informed consent. The study was conducted between August 2017 and September 2020 in Hong Kong, the Philippines, South Korea and Taiwan and each patient was followed for 1 year.

**FIGURE 1:**
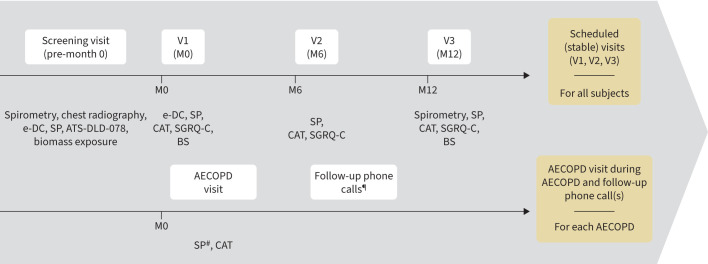
Study design. The four scheduled, stable-state chronic obstructive pulmonary disease (COPD) study visits were an initial screening visit (V0, pre-month 0), a baseline visit at month 0 (V1), a visit at month 6 (V2) and a final study visit at the end of the follow-up period at month 12 (V3). Additional unscheduled visits during acute exacerbations of COPD (AECOPD) were performed per protocol. From V0 to V1, eligible patients were trained in the use of the electronic diary card (e-DC) given to them at screening. At all study visits, sputum samples (SP) were collected for culture and PCR analyses for pathogen identification, and COPD Assessment Test (CAT) and St George's Respiratory Questionnaire for COPD (SGRQ-C) questionnaires were completed. ATS-DLD-078: American Thoracic Society and National Heart and Lung Institute Division of Lung Disease Respiratory Questionnaire; BS: blood sample. ^#^: sputum could be collected spontaneously or could be induced, as per the investigator's judgement; ^¶^: follow-up phone calls were made at least every 2 weeks until the exacerbation was resolved.

Four study visits were scheduled, with additional, unscheduled AECOPD visits performed within 96 h of the onset of an exacerbation (figure 1). The primary objective was to estimate the proportion of potential bacterial pathogens (by culture-based methods) and viral pathogens (by PCR) in the sputum of patients with stable COPD and at exacerbation over the course of 1 year. PCR estimation of the proportion of bacterial pathogens was assessed as a secondary objective. We also report results for secondary objectives relating to the incidence rate and duration of AECOPD and HRQOL, as well as descriptive results for tertiary objectives relating to the quantity (load) of specific bacteria and viruses. Estimates from a further analysis are also presented for the odds of experiencing an exacerbation (*versus* stable state) for the presence, acquisition and apparition of specific pathogens.

### Participants

Eligible patients were aged ≥40 years with a confirmed diagnosis of moderate to very severe stable COPD, with at least one documented exacerbation in the previous year requiring corticosteroids, antibiotics or hospitalisation. COPD was diagnosed based on post-bronchodilator spirometry, *i.e.* ratio of forced expiratory volume in 1 s (FEV_1_) to forced vital capacity <0.7, and FEV_1_ <80% predicted. Disease severity was classified as Global Initiative for Chronic Obstructive Lung Disease (GOLD) grade 2 (moderate), 3 (severe) or 4 (very severe) [1]. Patients were considered to have stable COPD if their last exacerbation episode had resolved within 30 days before study entry. Eligible patients had either a smoking history of at least 10 pack-years or a history of exposure to biomass smoke for at least 20 years and had to be able to provide a sputum sample.

Patients were excluded if they were diagnosed with a respiratory disorder other than COPD or had recent (≤3 months) chest radiography showing evidence of clinically significant abnormalities unrelated to COPD. Other exclusion criteria included α_1_-antitrypsin deficiency as an underlying cause of COPD, confirmed or suspected immunosuppression, lung surgery within 12 months before visit 1, chemotherapy within 12 months before visit 1, participation in another clinical study, antibiotic receipt within 1 month of study entry or continuous antibiotic administration (>30 days in total) within 90 days before visit 1 or systemic administration of corticosteroids (>14 days consecutive days) within 90 days before visit 1, and any condition that could interfere with the patient's ability to understand the study procedures. Pregnant women were not enrolled. Contraindications for spirometry testing were additional exclusion criteria.

### Exacerbation monitoring and sputum sample collection

The occurrence of a potential exacerbation was monitored using electronic diary cards to record morning symptoms each day. An exacerbation was defined as follows [25]: 1) worsening of two or more major symptoms (dyspnoea, sputum volume, sputum purulence) for two or more consecutive days; or 2) worsening of any major symptom together with any of the following minor symptoms for two or more consecutive days: sore throat, cold (nasal discharge and/or nasal congestion), fever (oral temperature ≥37.5°C) without other cause, increased cough, increased wheeze.

The exacerbation end date and severity were determined by a study qualified individual during follow-up phone calls, made at least every 2 weeks until the exacerbation was resolved. Exacerbation severity was classified as mild if controlled with an increased dosage of regular medications, moderate if requiring treatment with systemic corticosteroids and/or antibiotics, or severe if requiring hospitalisation.

Sputum samples were collected at all study visits (stable state and AECOPD) and were spontaneous or induced (using saline solution), as per the investigator's judgement. If an exacerbation occurred when a stable-state visit was planned, the visit was re-scheduled for when the patient had recovered. Spontaneous self-collection of sputum samples at the patient's home was allowed (only during exacerbation and if the patient needed an antibiotic dose before the AECOPD visit) but there were no cases of self-collection at home during the study. Patients were instructed not to take an antibiotic before study visits. However, in some instances, antibiotic treatment was already initiated. Data on the use of antibiotics before sputum collection were recorded at each study visit and at exacerbation.

### Laboratory assays

Sputum samples were diluted in dithiothreitol (DTT), processed and cultured within 6 h of collection for microbiology testing. Any remaining sample was stored at −70/80°C before shipment to the central study laboratory (Q^2^ Solutions, Singapore) for additional testing. Sputum sample quality was assessed by Gram stain, with no rejection criteria.

Bacterial pathogens were identified by standard bacteriological culture methods on fresh sputum samples at study site laboratories, according to each laboratory's routine methods. Bacterial species identified included Hi, Mcat, *Streptococcus pneumoniae*, *Staphylococcus aureus*, *Pseudomonas aeruginosa*, *Klebsiella pneumoniae* and *Acinetobacter baumannii*. Hi isolates were further analysed by PCR assay to identify non-typeable Hi or other *Haemophilus* species.

For PCR, nucleic acids were extracted using the MagNA Pure 96 equipment (Roche Diagnostics) with the DNA and Viral NA Large Volume Kit (Roche Diagnostics) and the Pathogen Universal protocol, as per the manufacturer's instructions. The presence of bacterial pathogens was assessed by PCR assay of frozen DTT-treated sputum samples in the central laboratory using two triplex real-time PCR assays; one quantitative, identifying Hi, Mcat and *S. pneumoniae*, and the other qualitative, identifying *Streptococcus pyogenes*, *S. aureus* and *P. aeruginosa*, as described previously [17] and summarised in the supplementary material. Viral pathogens were identified using a qualitative nucleic acid multiplex test and real-time PCR, as summarised in the supplementary material. Viral pathogens identified included HRV, respiratory syncytial virus, parainfluenza virus, enterovirus, metapneumovirus, influenza virus, adenovirus, bocavirus and coronavirus.

The concentration of bacterial or viral DNA (copies·mL^−1^) in each sample was inferred from a calibration curve made of serial dilutions of a plasmid containing the sequences targeted by the assay and converted from copies per PCR to copies·mL^−1^ of DTT-treated sputum samples.

### HRQOL assessment

Patients completed a self-administered COPD Assessment Test (CAT) at every study visit and self-administered St George's Respiratory Questionnaire for COPD patients (SGRQ-C) at each of the three scheduled study visits. For each questionnaire, higher scores reflect worse health status [26, 27].

### Statistical analysis

The aim was to enrol ∼200 patients. Assuming that ∼90% of these patients would have on average one AECOPD per year, of whom ∼80% would be able to provide one AECOPD sputum sample, ∼140 AECOPD sputum samples would be obtained. Assuming 20% of these samples were positive for Hi and Mcat, the exact 95% CI around this proportion was estimated as 13.7–27.6%, corresponding to 19–39 positive sputum samples, which was considered appropriate to address the study objectives.

The study objectives were assessed on eligible patients who fulfilled screening criteria and completed at least visit 1. Proportions of sputum samples positive for bacterial and viral pathogens and 95% CIs were calculated at stable and exacerbation visits, overall and for each species, using a generalised estimating equations model, assuming a binomial distribution for the response variable with logit as the link function and an exchangeable correlation structure to account for within-patient correlations. AECOPD incidence rates and 95% CIs were estimated using a generalised linear model, assuming a negative binomial distribution for the response variable with logarithm as the link function and the logarithm of follow-up time as an offset variable. If the negative binomial model did not converge due to underdispersion of data, a Poisson model was used to obtain both incidence rates and their 95% CIs. Incidence rates are presented as average number of exacerbations per person per year.

The odds of experiencing an exacerbation (*versus* being in a stable state) were calculated for 1) patients with sputum positive for Hi, Mcat or HRV *versus* those with negative sputum; 2) patients with sputum positive for Hi, Mcat or HRV for the first time during the study (acquisition) *versus* those without acquisition; and 3) patients with sputum positive for Hi, Mcat or HRV who were negative at the previous visit (apparition) *versus* those without apparition. To obtain the odds ratios (ORs) associated with these three analyses, a conditional logistic regression model, stratified by patient, was fitted. The model included the pathogen (positive or negative) and season (high season, October–March; low season, April–September) as independent variables.

Descriptive statistics were used to summarise demographic and disease characteristics and HRQOL scores. SAS Drug Development (SAS Institute Inc.) was used for all statistical analyses.

## Results

### Patients and sputum samples

**FIGURE 2:**
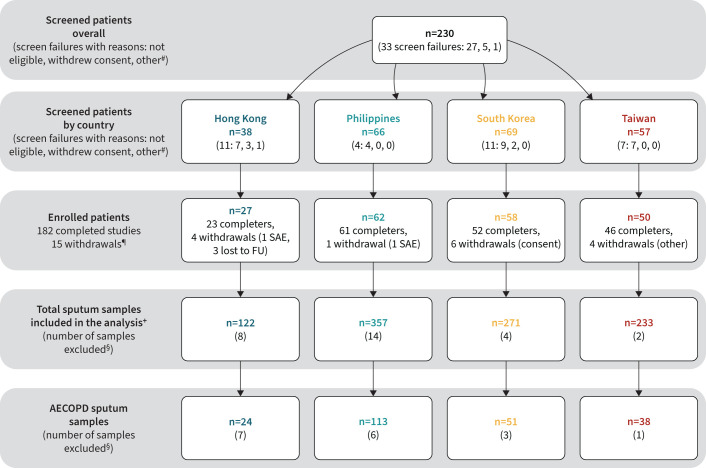
Patient disposition flow diagram. SAE: serious adverse event; FU: follow-up; AECOPD: acute exacerbations of chronic obstructive pulmonary disease. ^#^: other: patient experienced AECOPD during their screening visit or other reason not specified; ^¶^: withdrawals classified as consent withdrawal due to a SAE (SAE), consent withdrawal for another reason (consent), lost to follow-up before the final study visit (lost to FU), other (other); ^+^: includes samples obtained at all study visits (scheduled visits and unscheduled AECOPD visits); ^§^: sputum samples excluded from the full analysis set due to protocol deviations, patients had not recovered at a scheduled visit or samples taken from exacerbations occurring prior to visit 1.

Of 230 patients screened, 197 (85.7%) were enrolled and completed at least one visit; 182 (92.4%) completed the study (figure 2). In total, 983 sputum samples were obtained, 226 during AECOPD visits. Most samples were from the Philippines, with the fewest from Hong Kong (figure 2).

**FIGURE 3:**
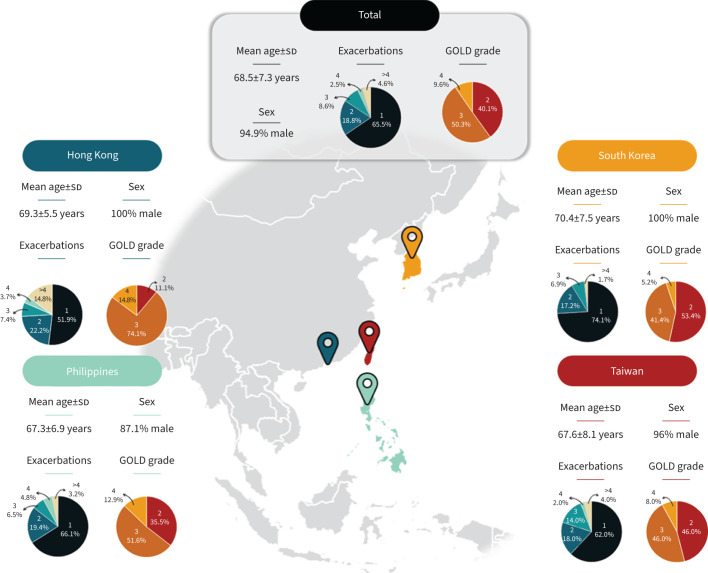
Patient baseline characteristics. Exacerbations are the number of exacerbations in the year before study inclusion. Global Initiative for Chronic Obstructive Lung Disease (GOLD) severity is graded as follows: 2=moderate airflow limitation, 3=severe airflow limitation, 4=very severe airflow limitation.

The mean age at screening was 68.5 years (range 67.3–70.4 years across countries; figure 3), 94.9% were male and patients had either East Asian (64%) or Southeast Asian ethnicity (36%). In the year before study enrolment, 129 patients (65.5%) had one exacerbation, 18.8% had two, 8.6% had three, 2.5% had four and 4.6% had more than four exacerbations (figure 3).

### AECOPD rate and characteristics

Overall, 227 exacerbations were recorded: 21 were mild, 182 moderate and 24 severe. The estimated yearly incidence rate of confirmed AECOPD (defined in table 1 footnote) was 1.27 per patient overall and increased with increasing GOLD grade at screening (table 1). The mean±sd duration of exacerbations overall was 14.3±9.0 days, with 20.3±11.1 days for mild, 12.6±6.3 days for moderate and 21.5±16.5 days for severe exacerbations.

**TABLE 1:** Estimated incidence rate of AECOPD during the 1-year follow-up

**GOLD grade at screening** ^#^	**Total subjects, n**	**Incidence rate**^¶^ **of AECOPD (95% CI)**	
		**Confirmed** ^+^	**Confirmed+potential** ^§^
**All grades**	197	1.27 (1.04–1.54)	1.30 (1.07–1.58)
**GOLD 2**	79	0.78 (0.54–1.14)	0.81 (0.56–1.16)
**GOLD 3**	99	1.51 (1.17–1.94)	1.56 (1.20–2.01)
**GOLD 4**	19	1.99 (1.37–2.89)	1.99 (1.37–2.89)

AECOPD: acute exacerbations of chronic obstructive pulmonary disease; GOLD: Global Initiative for Chronic Obstructive Lung Disease. ^#^: GOLD 2=moderate airflow limitation, GOLD 3=severe airflow limitation, GOLD 4=very severe airflow limitation; ^¶^: mean number of exacerbations per year from the negative binomial model (or Poisson model in cases of underdispersion); ^+^: confirmed AECOPD defined as exacerbation events plus missed exacerbation events (missed exacerbations correspond to all morning alerts confirmed by a phone call, as well as cases with no morning alert for which there was no site visit but for which AECOPD medical records were available), n=249; ^§^: potential AECOPD defined as all morning alerts confirmed by a phone call for which there was no site visit and for which no medical records were available, n=256.

### Prevalence of bacterial and viral species

**FIGURE 4:**
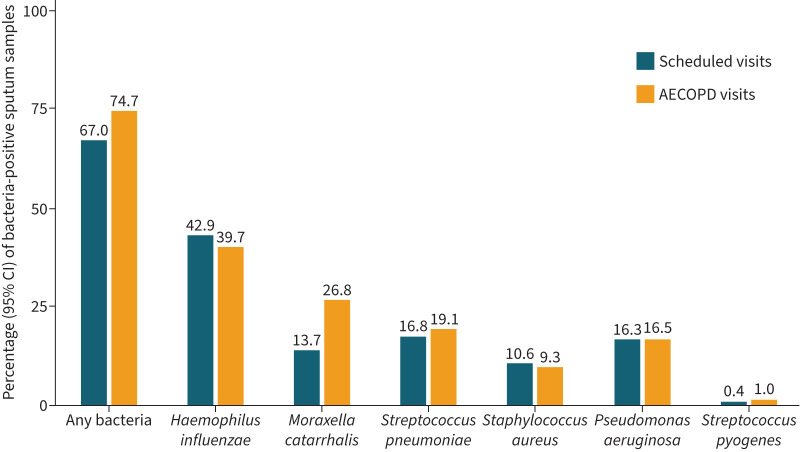
Percentage (95% CI) of sputum samples positive for bacteria by PCR analysis, by type of visit: scheduled or unscheduled acute exacerbation of chronic obstructive pulmonary disease (AECOPD) visit. Data from the culture analysis are provided in supplementary figure 1.

The culture and PCR results for percentages of sputum samples positive for specific bacterial species differed overall and by country (table 2, supplementary tables S1 and S2). Overall, the bacterial species detected most frequently by culture at stable state and exacerbation were *K. pneumoniae*, *P. aeruginosa* and Hi (table 2, supplementary figure S1). Hi was shown by subsequent PCR analysis to be mostly non-typeable (supplementary table S1). With PCR detection, the most prevalent bacteria detected were Hi, *S. pneumoniae* and *P. aeruginosa* at stable state and Hi, Mcat and *S. pneumoniae* at exacerbation (table 2, figure 4). *K. pneumoniae* presence was not assessed by PCR. The differences between culture and PCR detection results were likely due to better specificity and sensitivity with PCR than with culture [12, 17, 28, 29] and differences in routine culture-based methods used in this study between local laboratories. We therefore focus on the bacterial species results from the PCR assays, which were well characterised and conducted centrally.

**TABLE 2:** Bacterial pathogens detected in sputum samples by culture or PCR throughout study follow-up, during periods of stable disease (scheduled visits) and at exacerbation (AECOPD visits)

**Bacterial species**	**Percentage of bacteria-positive sputum samples (95% CI)**			
	**Culture**		**PCR**	
	**Scheduled visits** ^#^	**AECOPD visits** ^¶^	**Scheduled visits** ^+^	**AECOPD visits** ^§^
**Any**	74.2 (70.4–77.7)	78.0 (72.1–83.2)	67.0 (63.0–70.9)	74.7 (68.3–80.5)
***Haemophilus influenzae***	6.7	8.1	42.9 (38.7–47.0)	39.7 (33.0–46.7)
***Moraxella catarrhalis***	0.9 (0.3–1.9)	3.3 (1.5–6.4)	13.7 (11.0–16.8)	26.8 (20.9–33.3)
***Streptococcus pneumoniae***	2.5 (1.4–4.1)	2.4 (0.9–5.1)	16.8 (13.9–20.1)	19.1 (14.0–25.0)
***Streptococcus pyogenes***	NA	NA	0.4 (0.1–1.1)	1.0 (0.2–3.1)
***Staphylococcus aureus***	3.1 (1.8–4.7)	3.3 (1.5–6.4)	10.6 (8.2–13.4)	9.3 (5.7–13.9)
***Pseudomonas aeruginosa***	8.5 (6.4–11.0)	14.4 (10.0–19.5)	16.3 (13.4–19.6)	16.5 (11.7–22.1)
***Klebsiella pneumoniae***	26.4 (22.8–30.1)	24.9 (19.3–31.0)	NA	NA
***Acinetobacter baumannii***	2.2 (1.2–3.6)	4.3 (2.1–7.6)	NA	NA
**Other**	47.7 (43.5–51.8)	45.0 (38.3–51.8)	NA	NA

^#^: n=554; ^¶^: n=209; ^+^: n=546; ^§^: n=194. AECOPD: acute exacerbations of chronic obstructive pulmonary disease; NA: not assessed.

The analysis of prevalence data showed the proportion of sputum samples positive by PCR for any bacterial pathogen was higher at AECOPD visits than at stable-state visits (74.7% *versus* 67.0%, respectively), most notably for Mcat (26.8% in exacerbation *versus* 13.7% in stable state) (table 2, figure 4). The prevalence of Hi by PCR was similar between stable-state and exacerbation samples, overall and in each country (supplementary table S2). Sample positivity for Hi ranged from 28.9% (Taiwan) to 57.1% (Hong Kong) in stable-state samples, and from 16.2% (Taiwan) to 58.8% (Hong Kong) in exacerbation samples. Mcat prevalence ranged from 4.3% (Hong Kong) to 20.8% (Philippines) in stable-state samples, and from 18.9% (Taiwan) to 35.4% (Korea) in exacerbation samples. The prevalence of other bacterial species tended to be similar between stable state and exacerbation (figure 4, supplementary table S2).

Viral prevalence was low, other than for HRV (supplementary table S3). The proportion of sputum samples positive for viral pathogens was lower in stable-state samples than at exacerbation for HRV (8.1% and 16.6%, respectively), influenza virus (0.7% and 8.2%), coronavirus (2.6% and 5.7%) and parainfluenza virus (0.7% and 4.7%).

Analysis of bacterial load by quantitative PCR showed an approximate two-fold increase in mean±sd load for Hi at AECOPD visits (3.6×10^8^±11.3×10^8^ copies·mL^−1^ of sputum) *versus* stable state (1.6×10^8^±5.4×10^8^ copies·mL^−1^) and no difference between visit types for Mcat and *S. pneumoniae* (supplementary table S4). For HRV, there was an approximate two-fold increase in mean load at AECOPD visits (7.0×10^6^±18.7×10^6^ copies·mL^−1^) *versus* stable state (3.1×10^6^±13.9×10^6^ copies·mL^−1^) (supplementary table S4).

Analysis of the prevalence of bacterial pathogens in sputum samples according to the use of antibiotics *versus* no antibiotics before sputum sample collection suggested antibiotic administration had no major impact, although prior antibiotic administration was infrequent (supplementary table S5).

### Odds of exacerbation for pathogen presence, acquisition and apparition

**FIGURE 5:**
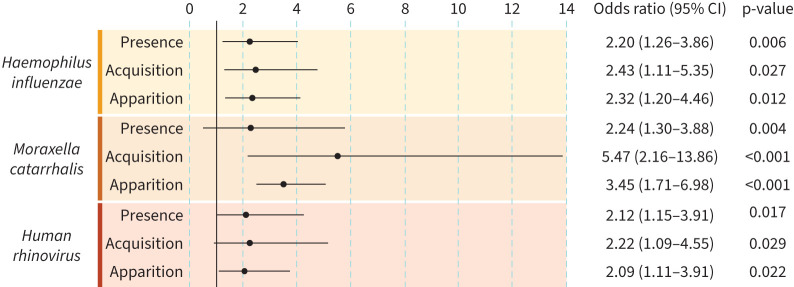
Odds ratios (95% CI) for effect of the presence, acquisition (sputum positive for the first time during the study) or apparition (sputum positive after negative sputum sample at previous visit) of *Haemophilus influenzae*, *Moraxella catarrhalis* or human rhinovirus detected by PCR on the odds of experiencing acute exacerbations of chronic obstructive pulmonary disease rather than being in stable state.

Using the PCR assay results, the odds of experiencing an exacerbation (*versus* stable state) was calculated according to pathogen detected as 1) presence *versus* absence; 2) acquisition (detection for the first time during the study) *versus* no acquisition; and 3) apparition (detection after negative sputum sample at previous visit) *versus* no apparition. The OR for experiencing an exacerbation rather than being in stable state was significant for Hi, Mcat and HRV detection for all three analyses (figure 5). Specifically, the odds of an exacerbation when there was presence, acquisition and apparition of Hi were 2.20 (95% CI 1.26–3.86, p=0.006), 2.43 (95% CI 1.11–5.35, p=0.027) and 2.32 (95% CI 1.20–4.46, p=0.012) times higher compared to no presence, no acquisition and no apparition of Hi, respectively. For Mcat, the ORs (95% CI) were 2.24 (1.30–3.88, p=0.004), 5.47 (2.16–13.86, p<0.001) and 3.45 (1.71–6.98, p<0.001) for presence, acquisition and apparition, respectively. For HRV, the ORs (95% CI) were 2.12 (1.15–3.91, p=0.017), 2.22 (1.09–4.55, p=0.029) and 2.09 (1.11–3.91, p=0.022) for presence, acquisition and apparition, respectively.

### HRQOL scores

**FIGURE 6:**
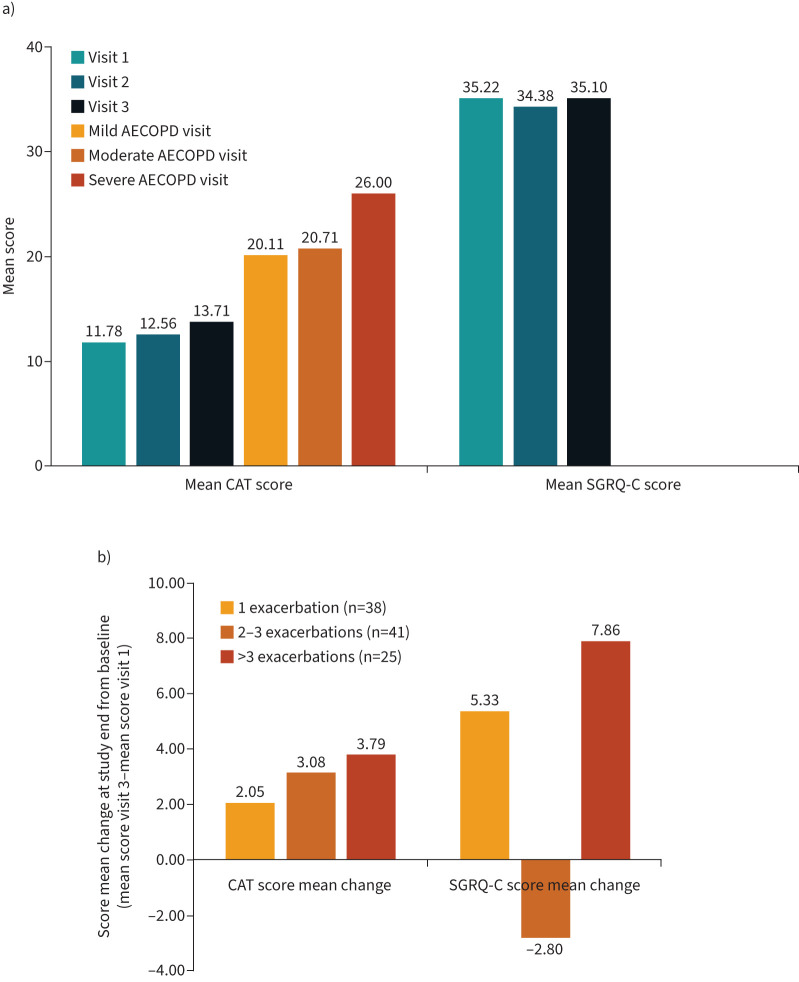
COPD Assessment Test (CAT) and St George's Respiratory Questionnaire for chronic obstructive pulmonary disease (SGRQ-C) scores by a) visit and b) by total score difference from the first to final visit according to number of exacerbations experienced during the 1-year follow-up. AECOPD: acute exacerbations of chronic obstructive pulmonary disease.

The mean CAT scores were 12.66 at stable-state visits, ∼20 at mild and moderate AECOPD visits and 26 at severe AECOPD visits (figure 6). Stable-state SGRQ-C scores remained constant over time (figure 6). Total CAT and SGRQ-C scores at the final scheduled visit were higher than those at the first scheduled visit for patients with more than three exacerbations compared with those experiencing fewer exacerbations during the 1-year follow-up (figure 6).

## Discussion

In this 1-year prospective follow-up study, we evaluated selected bacteria and viruses detected by culture-based methods and PCR in sputum samples from patients with COPD during periods of stable disease and exacerbation. We present findings from an analysis of the prevalence of each species, and an analysis of the odds of experiencing an exacerbation (*versus* stable-state COPD) for the presence, acquisition and apparition of specific pathogens.

Our results indicate higher sensitivity of PCR than culture assay in the identification of airway bacteria, as reported in AERIS and other studies of respiratory bacteria in patients with COPD [12, 17, 28, 29], with PCR positivity rates between five- and 15-fold higher than culture positivity for Hi and Mcat. As well as Hi and Mcat, other bacterial species identified most frequently by PCR assay were *S. pneumoniae* and *P. aeruginosa* at stable state and exacerbation, which is consistent with previous studies of patients with COPD [7, 23].

The proportion of sputum samples positive by PCR for any bacterial pathogen was higher at AECOPD visits than at stable state. However, examination of trends by species indicated a clear difference for Mcat only. The prevalence of all other species, including Hi, was similar in stable disease and at exacerbation. Analysis of detected viruses showed HRV was the most prevalent species and was overall twice as prevalent in AECOPD as in stable-state COPD. Another notable observation was that influenza virus was overall 12 times more prevalent in AECOPD than in stable state. This aligns with previous reports of sharp increases in viral prevalence from stable-state COPD to exacerbation state [7, 23]. Moreover, the OR for exacerbation (*versus* stable state) was significant when there was presence, acquisition or apparition of HRV, and for presence, acquisition and apparition of both Hi and Mcat. The OR results therefore indicate a potential involvement of Hi, Mcat and HRV in triggering an exacerbation. This was also suggested by results from the AERIS study, in which a significant OR for AECOPD occurrence was found when Mcat was detected, and a significant interaction was detected between non-typeable Hi and HRV presence and AECOPD risk [17].

Patients with severe and very severe COPD at screening had a higher incidence of exacerbations during the study than patients with moderate COPD, with exacerbation incidence rates ranging from under one per year (moderate, GOLD grade 2) to almost two per year (very severe, GOLD grade 4). A similar trend, but with lower AECOPD incidence rates, was reported in a retrospective analysis of 886 patients with COPD in the Netherlands, in which exacerbation incidence rates were two-fold higher in patients with very severe COPD than in those with moderate COPD [30]. We also found evidence of worsening general health status during the study that was more pronounced in patients experiencing more frequent exacerbations. Similar trends were observed in a meta-analysis of randomised trials involving over 18 000 patients with COPD, which showed incremental deteriorations in HRQOL with every moderate or severe COPD exacerbation over a 1-year period [31].

The patient population in this study was predominantly male and was of either East Asian or Southeast Asian heritage, potentially limiting the generalisability of our findings; however, this population was selected to address a data gap in the literature for patients with COPD. Another possible limitation is that, because of the smaller sample size, data from the Hong Kong cohort may be less robust. Also, as already highlighted, interpretation of the bacterial culture results is limited by the range of culture-based methods used in local laboratories. Finally, bacterial load may have a role in changing the clinical status of patients with COPD [32], but this was not analysed fully in this study. An important strength of the study is its prospective, longitudinal design that allowed collection of multiple sputum samples during periods of stable disease and exacerbation in the same individuals. All datasets and statistical analyses underwent double independent programming to ensure high quality data outputs.

### Conclusion

This study showed a high prevalence of Hi and Mcat bacteria in both stable and AECOPD states among patients with COPD in the Asia-Pacific region. Viral prevalence other than HRV was low, although influenza was markedly increased at exacerbation. The presence of Hi, Mcat and HRV significantly increased the odds of an exacerbation, providing further evidence of the potentially important role for these pathogens in exacerbations. Our results also confirm the negative impact of more frequent exacerbations on patients’ quality of life.

## Supplementary material

10.1183/23120541.00057-2022.Supp1 **Please note:** supplementary material is not edited by the Editorial Office, and is uploaded as it has been supplied by the author.
Supplementary material 00057-2022.SUPPLEMENT
