# Supplementary material for: Airway pathogens detected in stable and exacerbated COPD in patients in Asia-Pacific
Source: ERJ Open Res. 2022 Sep 26;8(3):00057-2022. doi: 10.1183/23120541.00057-2022 (PMC9511156; doi:10.1183/23120541.00057-2022)
Supplement: Supplementary file 1 [file 00057-2022.SUPPLEMENT.pdf]

Bacteria/viruses detected in moderate to very severe COPD and AECOPD in Asia-Pacific

Laura Taddei, Lucio Malvisi, David S. Hui, Ludovic Malvaux, Ronnie Z. Samoro, Sang Haak Lee, Yiu Cheong Yeung, Yu-Chih Liu, Ashwani Kumar Arora

Supplement

PCR methods

A triplex real-time quantitative PCR assay was used for the detection and quantification of the lipo-oligosaccharide glycosyltransferase encoding gene (*lgtC*) of *Haemophilus influenzae*, the CopB outer membrane protein encoding gene (*copB*) of *Moraxella catarrhalis* and the autolysin encoding gene (*lytA*) of *Streptococcus pneumoniae*. The presence of *Streptococcus pyogenes*, *Staphylococcus aureus* and *Pseudomonas aeruginosa* was determined using a qualitative triplex real-time PCR assay targeting conserved regions of the CDS23 gene, the clumping factor A encoding gene (*clfA*) and the GDP mannose dehydrogenase encoding gene (*algD*), respectively.

Isolates initially identified as *H. influenzae* by bacteriological methods were later retested by PCR, targeting the glycosyltransferase (*lgtC*) and outer membrane protein P6 (P6) encoding genes [1] to differentiate *H. influenzae* from *H. haemolyticus*.

The Seegene *Allplex* Respiratory Panel assays (Panels 1, 2 and 3) are qualitative multiplex one-step RT-PCR assays intended for the simultaneous detection and identification of multiple respiratory virus nucleic acids in respiratory specimens [2,3]. Panel 1 detects influenza A, including subtypes of influenza A (H1 and H3), and distinguishes between 2009 H1N1 and other H1N1 (seasonal) strains, influenza B and respiratory syncytial virus; panel 2 detects adenovirus, human metapneumovirus, parainfluenza virus 1–4 and enterovirus; panel 3 detects coronavirus (OC43, 229E, NL63), rhinovirus and bocavirus.

A quantitative real-time PCR assay was used for the detection and quantification of a fragment of a conserved region of the 5’ noncoding region of rhinovirus [4] in samples displaying a positive signal for rhinovirus/enterovirus by *Allplex* assay.

References

1. van den Bergh MR, Spijkerman J, Swinnen KM, et al. Effects of the 10-valent pneumococcal nontypeable *Haemophilus influenzae* protein D-conjugate vaccine on nasopharyngeal bacterial colonization in young children: a randomized controlled trial. *Clin Infect Dis*. 2013; 56: e30-39.

2. Lee J, Lee HS, Cho YG, et al. Evaluation of Allplex Respiratory Panel 1/2/3 multiplex real-time PCR assays for the detection of respiratory viruses with influenza A virus subtyping. *Ann Lab Med* 2018; 38: 46-50.

3. Huh HJ, Kim JY, Kwon HJ, et al. Performance evaluation of Allplex Respiratory Panels 1, 2, and 3 for detection of respiratory viruses and influenza A virus subtypes. *J Clin Microbiol* 2017; 55: 479-484.

4. Lu X, Holloway B, Dare RK, et al. Real-time reverse transcription-PCR assay for comprehensive detection of human rhinoviruses. *J Clin Microbiol* 2008; 46: 533-539.

Supplementary Table S1. Bacterial pathogens detected by culture throughout the entire study follow-up period in sputum samples during periods of stable disease and during AECOPD visits, overall and by country.

	**Percentage of pathogen-positive sputum samples (95% CI)***									
	**All**		**Hong Kong**		**Philippines**		**South Korea**		**Taiwan**	
	**Scheduled visits** N=554	**AECOPD visits** N=209	**Scheduled visits** N=70	**AECOPD visits** N=17	**Scheduled visits** N=179	**AECOPD visits** N=107	**Scheduled visits** N=161	**AECOPD visits** N=48	**Scheduled visits** N=144	**AECOPD visits** N=37
Any	74.2 (70.4-77.7)	78.0 (72.1-83.2)	97.1 (91.4-99.5)	94.1 (76.6-99.7)	87.7 (82.4-92.0)	90.7 (84.2-95.2)	48.4 (40.8-56.1)	56.2 (42.2-69.7)	75.0 (67.5-81.6)	62.2 (46.1-76.6)
Hi	6.7	8.1	5.7	29.4	5.6	6.5	8.7	6.3	6.3	5.4
NTHi	6.3	7.7	5.7	29.4	5.0	6.5	8.7	4.2	5.6	5.4
Non-Hi	0.4	0.5	0.0	0.0	0.6	0.0	0.0	2.1	0.7	0.0
M. catarrhalis	0.9 (0.3-1.9)	3.3 (1.5-6.4)	0.0 (0.0-5.1)	0.0 (0.0-19.5)	0.0 (0.0-2.0)	0.9 (0.1-4.1)	2.5 (0.8-5.7)	4.2 (0.7-12.3)	0.7 (0.0-3.0)	10.8 (3.5-23.4)
S. pneumoniae	2.5 (1.4-4.1)	2.4 (0.9-5.1)	1.4 (0.1-6.1)	0.0 (0.0-19.5)	0.6 (0.0-2.4)	0.0 (0.0-3.4)	6.2 (3.2-10.6)	8.3 (2.7-18.3)	1.4 (0.2-4.2)	2.7 (0.2-11.4)
S. aureus	3.1 (1.8-4.7)	3.3 (1.5-6.4)	0.0 (0.0-5.1)	0.0 (0.0-19.5)	2.2 (0.7-5.1)	1.9 (0.3-5.7)	0.0 (0.0-2.3)	0.0 (0.0-7.4)	9.0 (5.1-14.4)	13.5 (5.1-26.8)
P. aeruginosa	8.5 (6.4-11.0)	14.4 (10.0-19.5)	4.3 (1.1-10.7)	5.9 (0.3-23.4)	11.7 (7.6-17.0)	19.6 (12.9-27.8)	4.3 (1.9-8.2)	8.3 (2.7-18.3)	11.1 (6.7-16.9)	10.8 (3.5-23.4)
K. pneumoniae	26.4 (22.8-30.1)	24.9 (19.3-31.0)	1.4 (0.1-6.1)	0.0 (0.0-19.5)	38.5 (31.6-45.8)	36.4 (27.7-45.8)	19.9 (14.2-26.5)	12.5 (5.2-23.7)	30.6 (23.4-38.4)	18.9 (8.6-33.4)
A. baumannii	2.2 (1.2-3.6)	4.3 (2.1-7.6)	0.0 (0.0-5.1)	11.8 (2.1-32.1)	2.8 (1.0-5.9)	4.7 (1.7-9.8)	1.2 (0.2-3.8)	0.0 (0.0-7.4)	3.5 (1.3-7.3)	5.4 (0.9-15.8)
Other	47.7 (43.5-51.8)	45.0 (38.3-51.8)	88.6 (79.8-94.6)	70.6 (47.1-88.3)	61.5 (54.2-68.4)	57.0 (47.5-66.1)	23.0 (16.9-29.9)	31.3 (19.4-45.1)	38.2 (30.5-46.3)	16.2 (6.8-30.2)

AECOPD, acute exacerbations of chronic obstructive pulmonary disease; CI, confidence interval; Hi, *Haemophilus influenzae*; N, number of patients per given category; NTHi, non-typeable *Haemophilus influenzae*

*when computed

Supplementary Table S2 Bacterial pathogens detected by PCR throughout the entire study follow-up period in sputum samples collected during periods of stable disease (scheduled visits) and during AECOPD visits, overall and by country.

	**Percentage of pathogen-positive sputum samples (95% CI)**									
	**All**		**Hong Kong**		**Philippines**		**South Korea**		**Taiwan**	
	**Scheduled visits** N=546	**AECOPD visits** N=194	**Scheduled visits** N=70	**AECOPD visits** N=17	**Scheduled visits** N=173	**AECOPD visits** N=92	**Scheduled visits** N=161	**AECOPD visits** N=48	**Scheduled visits** N=142	**AECOPD visits** N=37
Any	67.0 (63.0-70.9)	74.7 (68.3-80.5)	68.6 (57.2-78.6)	88.2 (67.9-97.9)	73.4 (66.5-79.6)	75.0 (65.5-83.1)	70.2 (62.8-76.9)	83.3 (71.2-92.0)	54.9 (46.7-63.0)	56.8 (40.7-71.9)
H. influenzae	42.9 (38.7-47.0)	39.7 (33.0-46.7)	57.1 (45.4-68.3)	58.8 (35.4-79.7)	46.8 (39.5-54.3)	44.6 (34.7-54.8)	44.7 (37.2-52.4)	41.7 (28.4-55.8)	28.9 (21.8-36.7)	16.2 (6.8-30.2)
M. catarrhalis	13.7 (11.0-16.8)	26.8 (20.9-33.3)	4.3 (1.1-10.7)	23.5 (8.0-46.5)	20.8 (15.2-27.3)	26.1 (17.9-35.6)	15.5 (10.5-21.6)	35.4 (22.9-49.5)	7.7 (4.1-12.9)	18.9 (8.6-33.4)
S. pneumoniae	16.8 (13.9-20.1)	19.1 (14.0-25.0)	4.3 (1.1-10.7)	5.9 (0.3-23.4)	20.2 (14.7-26.6)	21.7 (14.2-30.9)	26.7 (20.3-33.9)	27.1 (15.9-40.6)	7.7 (4.1-12.9)	8.1 (2.1-19.7)
S. aureus	10.6 (8.2-13.4)	9.3 (5.7-13.9)	5.7 (1.8-12.8)	5.9 (0.3-23.4)	11.6 (7.4-16.9)	8.7 (4.1-15.6)	9.9 (6.0-15.2)	10.4 (3.9-21.1)	12.7 (7.9-18.8)	10.8 (3.5-23.4)
P. aeruginosa	16.3 (13.4-19.6)	16.5 (11.7-22.1)	15.7 (8.5-25.4)	5.9 (0.3-23.4)	19.7 (14.2-26.0)	16.3 (9.7-24.7)	10.6 (6.4-15.9)	14.6 (6.5-26.3)	19.0 (13.1-26.0)	24.3 (12.5-39.6)
S. pyogenes	0.4 (0.1-1.1)	1.0 (0.2-3.1)	0.0 (0.0-5.1)	0.0 (0.0-19.5)	1.2 (0.2-3.5)	2.2 (0.4-6.6)	0.0 (0.0-2.3)	0.0 (0.0-7.4)	0.0 (0.0-2.6)	0.0 (0.0-9.5)

AECOPD, acute exacerbations of chronic obstructive pulmonary disease; CI, confidence interval; Hi, *Haemophilus influenzae*; N, number of patients per given category; NTHi, non-typeable *Haemophilus influenzae*; PCR, polymerase chain reaction

Supplementary Table S3. Viral pathogens detected by PCR throughout the entire study follow-up period in sputum samples collected during periods of stable disease (scheduled visits) and during AECOPD visits, overall and by country.

	**Percentage of pathogen-positive sputum samples (95% CI)**									
	**All**		**Hong Kong**		**Philippines**		**South Korea**		**Taiwan**	
	**Scheduled visits** N=546	**AECOPD visits** N=194	**Scheduled visits** N=70	**AECOPD visits** N=17	**Scheduled visits** N=173	**AECOPD visits** N=92	**Scheduled visits** N=161	**AECOPD visits** N=48	**Scheduled visits** N=142	**AECOPD visits** N=37
Any	15.0 (12.2-18.2)	35.6 (29.1-42.5)	12.9 (6.4-22.0)	35.3 (15.8-58.9)	24.3 (18.3-31.0)	40.2 (30.6-50.4)	11.2 (6.9-16.7)	33.3 (21.1-47.3)	9.2 (5.1-14.6)	27.0 (14.6-42.6)
RSV	0.9 (0.3-2.0)	1.0 (0.2-3.1)	0.0 (0.0-5.1)	0.0 (0.0-19.5)	1.7 (0.4-4.4)	1.1 (0.1-4.7)	1.2 (0.2-3.8)	0.0 (0.0-7.4)	0.0 (0.0-2.6)	2.7 (0.2-11.4)
Parainfluenza virus	0.7 (0.2-1.7)	4.7 (2.3-8.2)	0.0 (0.0-5.1)	5.9 (0.3-23.4)	1.2 (0.2-3.5)	2.2 (0.4-6.6)	0.6 (0.0-2.7)	2.1 (0.1-9.0)	0.7 (0.0-3.1)	13.5 (5.1-26.8)
Enterovirus	0.6 (0.1-1.4)	0.0 (0.0-1.9)	0.0 (0.0-5.1)	0.0 (0.0-19.5)	1.7 (0.4-4.4)	0.0 (0.0-3.9)	0.0 (0.0-2.3)	0.0 (0.0-7.5)	0.0 (0.0-2.6)	0.0 (0.0-9.5)
HRV	8.1 (6.0-10.6)	16.6 (11.8-22.3)	8.6 (3.5-16.6)	17.6 (4.7-39.6)	13.9 (9.3-19.5)	18.5 (11.5-27.2)	5.0 (2.3-9.1)	17.0 (8.2-29.4)	4.3 (1.7-8.4)	10.8 (3.5-23.4)
Metapneumovirus	0.0 (0.0-0.7)	0.0 (0.0-1.9)	0.0 (0.0-5.1)	0.0 (0.0-19.5)	0.0 (0.0-2.1)	0.0 (0.0-3.9)	0.0 (0.0-2.3)	0.0 (0.0-7.5)	0.0 (0.0-2.6)	0.0 (0.0-9.5)
Influenza virus	0.7 (0.2-1.7)	8.2 (4.9-12.7)	1.4 (0.1-6.1)	5.9 (0.3-23.4)	0.6 (0.0-2.5)	8.7 (4.1-15.6)	0.6 (0.0-2.7)	8.3 (2.7-18.3)	0.7 (0.0-3.1)	8.1 (2.1-19.7)
Adenovirus	2.0 (1.1-3.4)	4.1 (1.9-7.6)	1.4 (0.1-6.1)	0.0 (0.1-19.5)	1.7 (0.4-4.4)	6.5 (2.6-12.8)	1.9 (0.5-4.8)	2.1 (0.1-9.0)	2.8 (0.9-6.5)	2.7 (0.2-11.4)
Bocavirus	0.2 (0.0-0.8)	0.0 (0.0-1.9)	0.0 (0.0-5.1)	0.0 (0.0-19.5)	0.6 (0.0-2.5)	0.0 (0.0-3.9)	0.0 (0.0-2.3)	0.0 (0.0-7.4)	0.0 (0.0-2.6)	0.0 (0.0-9.5)
Coronavirus	2.6 (1.5-4.1)	5.7 (3.0-9.5)	2.9 (0.5-8.6)	5.9 (0.3-23.4)	4.6 (2.1-8.4)	8.7 (4.1-15.6)	1.9 (0.5-4.8)	4.2 (0.7-12.3)	0.7 (0.0-3.1)	0.0 (0.0-9.5)

AECOPD, acute exacerbations of chronic obstructive pulmonary disease; CI, confidence interval; HRV, human rhinovirus; N, number of patients per given category; PCR, polymerase chain reaction; RSV, respiratory syncytial virus

Supplementary Table S4. Bacterial and viral load results by quantitative PCR at scheduled stable-state or exacerbation visits.

	Mean load, copies/mL sputum (SD)	
**Species**	**Scheduled visits (N=554)**	**AECOPD visits (N=209)**
H. influenzae	1.6×10^8 (5.4×10^8)	3.6×10^8 (11.3×10^8)
M. catarrhalis	1.9×10^8 (7.3×10^8)	1.8×10^8 (2.7×10^8)
S. pneumoniae	1.0×10^8 (3.1×10^8)	1.2×10^8 (2.3×10^8)
HRV	3.1×10^6 (13.9×10^6)	7.0×10^6 (18.7×10^6)

AECOPD, acute exacerbations of chronic obstructive pulmonary disease; HRV, human rhinovirus; N, number of sputum samples per given category; SD, standard deviation

Supplementary Table S5. Prevalence of bacteria (no bacteria or any bacteria) by culture and PCR at scheduled stable-state or exacerbation visits according to antibiotic administration before sputum sample collection.

	**Scheduled visits**			**AECOPD visits**		
	**Number of sputum samples**	**No bacteria, N (%)**	**Any bacteria, N (%)**	**Number of sputum samples**	**No bacteria, N (%)**	**Any bacteria, N (%)**
Culture						
No antibiotic administered	553	142 (25.7)	411 (74.3)	195	41 (21.0)	154 (79.0)
Antibiotic administered	1	1 (100)	0 (0)	14	5 (35.7)	9 (64.3)
PCR						
No antibiotic administered	545	179 (32.8)	366 (67.2)	180	46 (25.6)	134 (74.4)
Antibiotic administered	1	1 (100)	0 (0)	14	3 (21.4)	11 (78.6)

AECOPD, acute exacerbations of chronic obstructive pulmonary disease; N, number of sputum samples per given category

Supplementary Figure S1. Percentage (95% confidence intervals) of sputum samples positive for bacteria by culture analysis, by type of visit: scheduled or unscheduled AECOPD visit**.**

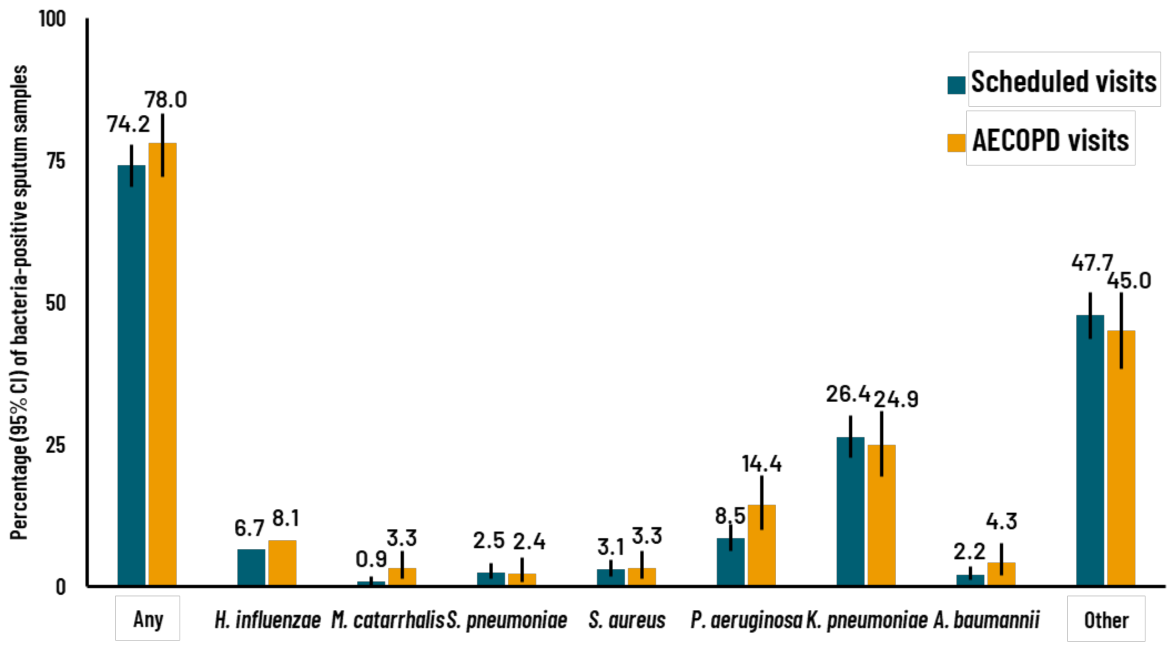

AECOPD, acute exacerbations of chronic obstructive pulmonary disease; CI, confidence interval
